# Impact of multiple drug-resistant Gram-negative bacterial bacteraemia on infected pancreatic necrosis patients

**DOI:** 10.3389/fcimb.2022.1044188

**Published:** 2022-11-24

**Authors:** Di Wu, Yan Jia, Wenhao Cai, Yilin Huang, Arjun Kattakayam, Diane Latawiec, Robert Sutton, Jie Peng

**Affiliations:** ^1^ Department of Gastroenterology, Xiangya Hospital, Central South University, Changsha, China; ^2^ Liverpool Pancreatitis Research Group, Institute of Systems, Molecular and Integrative Biology, University of Liverpool and Liverpool University Hospitals NHS Foundation Trust, Liverpool, Merseyside, United Kingdom; ^3^ West China Centre of Excellence for Pancreatitis, Institute of Integrated Traditional Chinese and Western Medicine, West China-Liverpool Biomedical Research Centre, West China Hospital, Sichuan University, Chengdu, China

**Keywords:** multiple drug-resistant, Gram-negative bacterial bacteraemia, infected pancreatic necrosis, acute pancreatitis, mortality

## Abstract

**Introduction:**

Multiple drug-resistant Gram-negative bacterial (MDR-GNB) bacteraemia poses a serious threat to patients in hospital. Infected pancreatic necrosis (IPN) patients are a vulnerable population to infectious complications during hospitalization. This study aims to evaluate the impact of MDR Gram-negative bacteraemia on IPN patients.

**Methods:**

A case–control study was performed with data collected from 1 January 2016 to 1 July 2022 in a Chinese tertiary teaching hospital. Clinical data of the IPN patients with MDR-GNB bacteraemia were analyzed and compared to those of a matched control group without MDR-GNB bacteraemia (case–control ratio of 1:2). Comparisons were performed between with/without MDR-GNB bacteraemia and different severities of acute pancreatitis (AP). Independent predictors of overall mortality were identified *via* univariate and multivariate binary logistic regression analyses.

**Results:**

MDR-GNB bacteraemia was related to a higher mortality rate (62.5% vs. 8.3%, *p* < 0.001). Severe AP combined with MDR-GNB bacteraemia further increased mortality up to 81.3% (*p* = 0.025). MDR-GNB bacteraemia (odds ratio (OR) = 8.976, 95% confidence interval (CI) = 1.805 –44.620, *p* = 0.007) and severe AP (OR = 9.414, 95% CI = 1.742 –50.873, *p* = 0.009) were independent predictors of overall mortality. MDR- *Klebsiella pneumoniae* was the most common causative pathogen.

**Conclusion:**

A higher mortality rate in IPN patients was related to MDR-GNB bacteraemia and further increased in severe AP patients combined with MDR-GNB bacteraemia.

## Introduction

Acute pancreatitis (AP) is an unpredictable and potentially lethal disease of the gastrointestinal system with increasing occurrence within the past decade (Boxhoorn et al., 2020; Szatmary et al., 2022). Approximately 30% of AP patients will develop severe acute pancreatitis (SAP) and infected pancreatic necrosis (IPN), which are respectively the major causes for the first and second peaks of mortality in AP (Brown et al., 2014; Werge et al., 2016; Baron et al., 2020).

Pancreatic necrosis is sterile in the early disease stages, but as many as 30% of cases will develop into IPN as the disease progresses (Garret et al., 2020). Gram-negative bacteria account for more than half of all bacterial infections in hospitalized patients, including AP patients. Specifically, moderately SAP and SAP patients are the most susceptible populations to multiple drug-resistant Gram-negative bacterial (MDR-GNB) infections due to prolonged hospitalization and frequent use of invasive interventions and antibiotics (Diekema et al., 2019; Ning et al., 2021b). The overuse of antibiotics for AP patients in China, which ranks first in the world over the most recent decade, may be the main reason for MDR-GNB infections (Parniczky et al., 2019).

The emergence of MDR-GNB infections with increasing prevalence merits more attention because limited antibiotic treatment choices for MDR-GNB strains result in a greater potential of developing severe sepsis (Wolbrink et al., 2020). The impact of MDR-GNB infectious complications on the outcomes of AP patients is not consistent between different centers in other countries (Jain et al., 2018; Shi et al., 2020). Some studies have highlighted an increase in the risk of MDR bacterial infections among AP patients, which aimed to differentiate between MDR and non-MDR bacterial infections but failed to investigate the role of MDR-GNB bacteraemia (Moka et al., 2018; Ning et al., 2019; Garret et al., 2020; Li et al., 2020). Other studies have revealed that organ failure (OF) and IPN may be two independent risk factors for a poor AP prognosis, but IPN has been regarded as less important than organ failure (Shi et al., 2020; Windsor and de-Madaria, 2021). An infectious complication of AP cannot be arbitrarily defined as IPN, and it also needs to include bacteraemia or pulmonary infection (Wu et al., 2022a). Moreover, whilst it is often overlooked, bacteraemia, especially MDR-GNB bacteraemia, may pose a serious threat to IPN patients (Ning et al., 2021a). MDR-GNB bacteraemia mortalities have been reported in several studies of hospital- onset infections, but they still have not been fully addressed in IPN patients (Tsai et al., 2014). In addition, given clear MDR-GNB differences and the many variables associated with adverse outcomes, it may be more useful to investigate the mortality rates in IPN patients with MDR-GNB bacteraemia.

Currently, no report exists that focuses on the MDR-GNB bacteraemia in IPN patients, which suggests that large gaps remain in this area. The purpose of the present study was as follows: 1) to investigate the role of MDR-GNB bacteraemia in IPN patients, 2) to determine the relationship between AP severity and MDR-GNB bacteraemia, 3) to identify the independent predictors of mortality among IPN patients, and 4) to describe the distribution of MDR-GNB strains in IPN patients. Our findings may shed light on the improvement of treatment and prognosis among this population.

## Methods

### Study design, setting, and ethics

**Figure 1:**
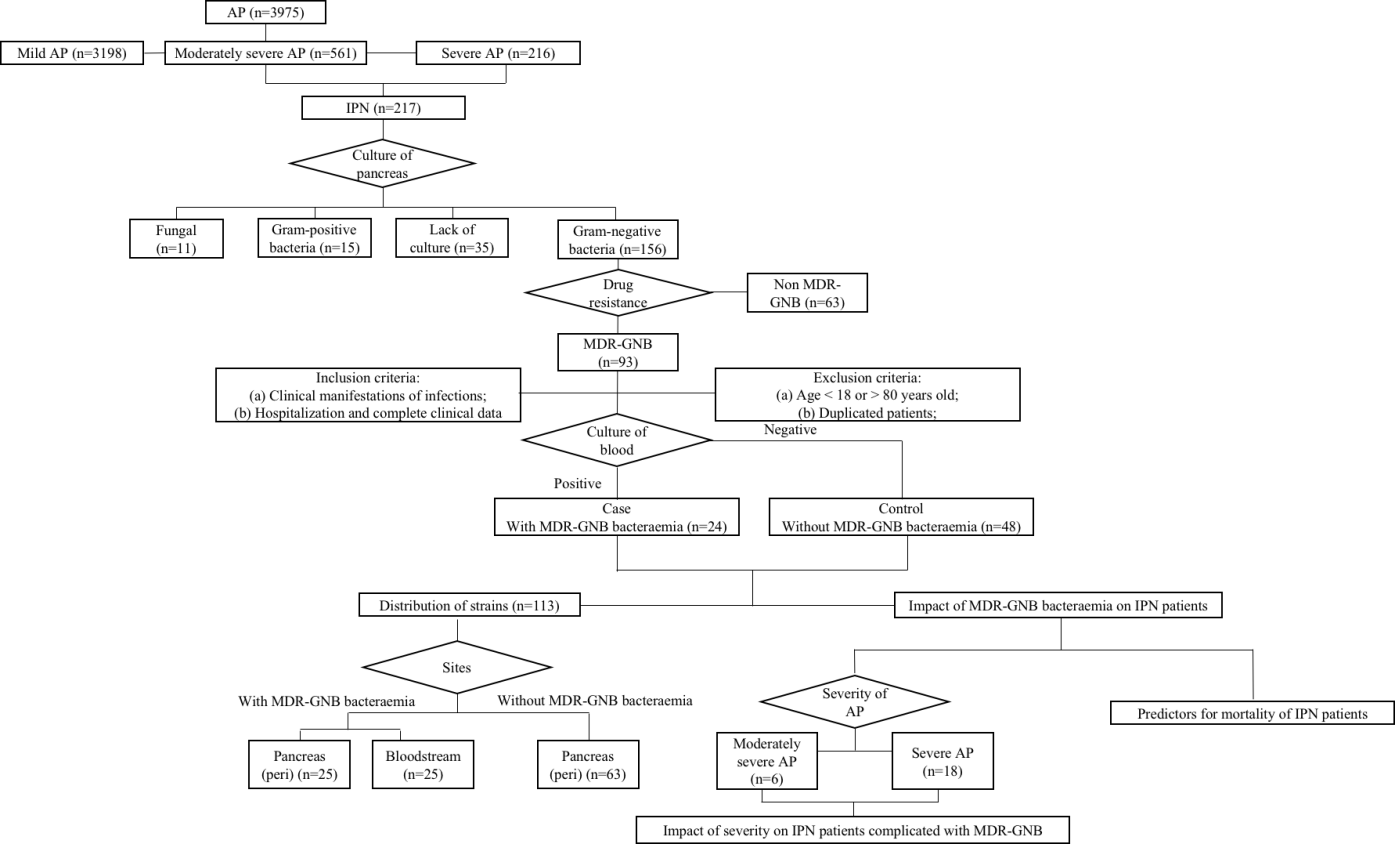
Flow chart of the study.

This retrospective study was performed at Xiangya Hospital, a 3,500-bed tertiary-care teaching hospital, affiliated with Central South University, Changsha, Hunan, China. Data from the electronic patient record system of IPN patients with and without MDR-GNB bacteraemia, from 1 February 2016 to 1 July 2022, were collected. A case–control study with a case–control ratio of 1:2 was performed. The control group was age ( ± 2) and gender- matched MDR-GNB IPN patients without MDR-GNB bacteraemia. IPN patients with missing data or without positive MDR-GNB culture at the pancreas (**Figure 1**) were excluded. The clinical characteristics included severity, aetiology, age, sex, referral timing from the onset of AP, length of hospitalization including also intensive care unit (ICU) stay, bedside index of severity in acute pancreatitis (BISAP) at admission, major complications, mortality, and laboratory variables at the onset of MDR-GNB bacteraemia. The role of both severity and MDR-GNB bacteraemia among IPN patients, predictors of overall mortality, and distribution of causative pathogens were investigated. This study was approved by the Institutional Review Board of Xiangya Hospital (No. 202103047). Informed consent was waived due to the study’s retrospective nature, and confidentiality was assured throughout the study.

### Definitions

The presence of bacteraemia was diagnosed based on the clinical manifestations and positive results of specimens according to the criteria of the Centers for Disease Control (CDC) (Horan et al., 2008; Wu et al., 2021a). The onset of MDR-GNB bacteraemia was defined as the collection date of the first positive culture for blood, which was divided into three time periods including pre-intervention and post-intervention (≤5 and >5 days, respectively) (Carey et al., 2011). The diagnosis, severity, and classification of AP followed the criteria of the Revised Atlanta Classification: 1) mild AP: the absence of either OF or local/systemic complications; 2) moderately SAP: the presence of transient OF (less than 48 h) and/or local or systemic complications; 3) SAP: the presence of persistent OF (more than 48 h) (Banks et al., 2013). OF was defined for the three organ systems (respiratory, cardiovascular, and renal) according to the modified Marshall Score (Marshall et al., 1995). IPN was defined as peri-pancreatic specimens obtained with positive MDR-GNB culture during the first intervention of pancreatic necrosis. The criteria of AP aetiology were as follows: 1) biliary: radiological evidence of abdominal ultrasonography with increased serum alanine aminotransferase; 2) alcoholism: regular drink over 50 g/day; 3) hypertriglyceridemia: triglycerides of more than 5.6 mmol/L without any other clear aetiology. Gram-positive bacterial or fungal infections were defined as the presence of concomitant infections (Wu et al., 2022b). MDR- GNB is defined as strains not sensitive to three or more categories of antibiotics (Wu et al., 2021b). Combination antibiotic therapy was defined as a regimen with at least two categories of antibiotics based on the condition of patients and approved by infectious disease specialists.

### Patients, management, and microbiology

All patients were assessed and managed at admission according to the latest international guidelines *via* the multi-disciplinary team (Crockett et al., 2018). The antibiotic regimen we recorded was the initial therapy (3–5 days) after obtaining the culture results, which could potentially change or be combined with new antibiotics, dependent on whether the patients’ condition worsened. If there was an AP patient with suspected bacteraemia, we would perform a blood culture in accordance with the standard protocols from the CDC (Horan et al., 2008). A conventional blood culture set consists of aerobic and anaerobic bottles. A collection of 20–30 ml of blood requires more than two bottles and is obtained within a few hours of each other *via* peripheral venipuncture when obtaining blood cultures for a total volume of 40–60 ml of blood to optimize the detection of pathogens (Doern et al., 2019). Identification and drug-resistant test of MDR-GNB were performed *via* the Vitek-2 system and broth micro-dilution method, respectively (Wu et al., 2021a).

### Statistical analysis

The continuous variables expressed using medians with standard deviations were compared with Student’s t- test, Welch’s t- test, Mann–Whitney U test, or Fisher’s exact test, as appropriate. Categorical variables described in absolute numbers and percentages were compared with the χ^2^ test or Fisher’s exact tests. The binary logistic regression analysis was used to determine independent predictors of mortality *via* univariate and multivariate analyses. The odds ratio (OR) and 95% confidence interval (CI) were calculated to evaluate the associations. *p*-Value < 0.05 (two-tailed) was considered statistically significant. Statistical analyses were performed using SPSS 26.0.

## Results

### Clinical characteristics and outcome of infected pancreatic necrosis patients

As seen in **Table 1**, 56 patients (77.8%) were male with a mean age of 46.8 ± 11.4 years. Hypertriglyceridemia (n = 32, 44.4%) was the leading aetiology of total patients; however, biliary source is significantly higher in the MDR-GNB bacteraemia group when compared to the contrast group (45.8% vs. 18.8%, *p* = 0.027). The MDR-GNB bacteraemia group showed significantly longer ICU stays (21.6 days vs. 4.1 days, *p* < 0.001) and a higher BISAP score at admission (3.0 vs. 2.1, *p* = 0.001) than the contrast group. SAP (66.7% vs. 25.0%, *p* < 0.001) more frequently occurred in the MDR-GNB bacteraemia group. The mortality rate was significantly higher among patients with MDR-GNB bacteraemia than those without MDR-GNB bacteraemia infections (62.5% vs. 8.3%, *p* < 0.001).

**Table 1:** Clinical characteristics and comparison between IPN patients with and without MDR-GNB bacteraemia.

Characteristics	Total	Control, without MDR-GNB bacteraemia (n = 48)	Case, with MDR-GNB bacteraemia (n = 24)	*p*-Value
Age, years (mean ± SD)	46.8 ± 11.4	45.8 ± 10.9	49.0 ± 12.4	0.253
Sex, n (%)				0.841
Male	56 (77.8)	37 (77.1)	19 (79.2)	
Female	16 (22.2)	11 (22.9)	5 (20.8)	
Classification of AP, n (%)				<0.001*
Moderately SAP	44 (61.1)	36 (75.0)	8 (33.3)	
SAP	28 (38.9)	12 (25.0)	16 (66.7)	
Aetiology, n (%)				0.027*
Hypertriglyceridemia	32 (44.4)	21 (43.8)	11 (45.8)	
Biliary	20 (27.8)	9 (18.8)	11 (45.8)	
Alcoholism	4 (5.6)	4 (8.3)	0	
Others	16 (22.2)	14 (29.2)	2 (8.2)	
Recurrent AP, n (%)	11 (15.3)	6 (12.5)	5 (20.8)	0.563
Concomitant infections, n (%)	18 (25.0)	11 (22.9)	7 (29.2)	0.564
Referred patient, n (%)	68 (94.4)	45 (93.8)	23 (95.8)	0.998
Intensive care units stay, days (mean ± SD)	9.9 ± 13.8	4.1 ± 4.8	21.6 ± 18.0	<0.001*
Hospitalization, days (mean ± SD)	40.8 ± 22.1	37.6 ± 19.3	47.1 ± 26.0	0.085
Intervention, n (%)				0.067
PCD	8 (11.1)	5 (10.4)	3 (12.5)	
PCD to minimal access retroperitoneal necrosectomy	56 (77.8)	41 (85.4)	15 (62.5)	
OPN	2 (2.8)	0	2 (8.3)	
Step-up to OPN	6 (8.3)	2 (4.2)	4 (16.7)	
BISAP score at admission (mean ± SD)	2.4 ± 1.0	2.1 ± 0.8	3.0 ± 1.1	0.001*
Details of BISAP score, n (%)				
Blood urea nitrogen >25 mg/dl	27 (37.5)	11 (22.9)	16 (66.7)	<0.001*
Impaired mental status	8 (11.1)	0	8 (33.3)	<0.001*
Age > 60	12 (16.7)	7 (14.6)	5 (20.8)	0.737
Pleural effusion present	65 (90.3)	44 (91.7)	21 (87.5)	0.769
SIRS criteria (>2)	60 (83.3)	38 (79.2)	22 (91.7)	0.314
Major complications, n (%)				
Hemorrhage	17 (23.6)	8 (16.7)	9 (37.5)	0.051
Intestinal leakage	10 (13.9)	3 (6.3)	6 (25.0)	0.059
Pancreatic fistula	11 (15.3)	5 (10.4)	6 (25.0)	0.203
Mortality	19 (26.4)	4 (8.3)	15 (62.5)	<0.001*

Note. SD, standard deviation; PCD, percutaneous catheter drainage; OPN, open necrosectomy; SIRS, systemic inflammatory response syndrome; MDR-GNB, multiple drug-resistant Gram-negative bacterial; IPN, infected pancreatic necrosis; AP, acute pancreatitis; SAP, severe acute pancreatitis; BISAP, bedside index of severity in acute pancreatitis.

*p-Values are statistically significant between with and without MDR-GNB bacteraemia groups.

### Comparison between moderately severe acute pancreatitis and severe acute pancreatitis among multiple drug-resistant Gram-negative bacterial bacteraemia group

In **Table 2**, MDR-GNB bacteraemia was observed in eight moderately SAP and 16 SAP patients. SAP patients showed a significantly higher procalcitonin level when compared to the contrast group (35.5 vs. 5.1 ng/L, *p* = 0.020). Inappropriate empirical therapy was not significantly different between the moderately SAP and SAP groups (37.5% vs. 31.3%, *p* = 0.761). The mortality rate was significantly higher in the SAP group when compared with the moderately SAP group (81.3% vs. 25.0%, *p* = 0.025). There were three and nine patients diagnosed with bacteraemia during the post-intervention (≤5 days) for IPN with no significant difference in the moderately SAP and SAP groups, respectively (*p* = 0.096).

**Table 2:** Clinical characteristics and comparison between different categories among 24 IPN patients with MDR-GNB bacteraemia.

Characteristics	Moderately SAP (n = 8)	SAP (n = 16)	*p*-Value
Age, years (mean ± SD)	53.3 ± 13.9	46.9 ± 11.4	0.247
Sex, n (%)			1.000
Male	6 (75.0)	13 (81.2)	
Female	2 (25.0)	3 (18.8)	
Aetiology, n (%)			0.113
Hypertriglyceridemia	6 (75.0)	5 (31.3)	
Biliary	2 (25.0)	9 (56.2)	
Others	0	2 (12.5)	
Concomitant infections, n (%)	3 (37.5)	6 (37.5)	1.000
Onset of bacteraemia, n (%)			0.096
Pre-intervention	0	3 (18.8)	
Post-intervention (≤5 days)	3 (37.5)	9 (56.2)	
Post-intervention (> 5 days)	5 (62.5)	4 (25.0)	
Intervention, n (%)			0.789
PCD	1 (12.5)	2 (12.5)	
PCD—minimal access retroperitoneal necrosectomy	4 (50.0)	11 (68.8)	
OPN	1 (12.5)	1 (6.2)	
Step-up to OPN	2 (25.0)	2 (12.5)	
Laboratory and clinical variables (mean ± SD)			
Albumin, g/L	29.8 ± 3.2	30.6 ± 4.0	0.631
Neutrophil count, 10^3^/mm^3^	7.2 ± 4.6	12.3 ± 9.1	0.152
Lymphocyte count, 10^3^/mm^3^	0.8 ± 0.7	1.0 ± 1.6	0.807
Procalcitonin, ng/L	5.1 ± 5.2	35.5 ± 46.2	0.020*
Highest temperature, °C	39.2 ± 0.7	39.0 ± 1.0	0.690
Inappropriate empirical therapy	3 (37.5)	5 (31.3)	0.761
Antibiotic therapy, n (%)			0.885
Monotherapy	5 (62.5)	8 (50.0)	
Polytherapy	3 (37.5)	8 (50.0)	
Mortality, n (%)	2 (25.0)	13 (81.3)	0.025*

IPN, infected pancreatic necrosis; MDR-GNB, multiple drug-resistant Gram-negative bacterial; SAP, severe acute pancreatitis; PCD, percutaneous catheter drainage; OPN, open necrosectomy.

*p-Values are statistically significant between moderately SAP and SAP groups among MDR-GNB bacteraemia patients.

### Predictors of mortality

The association of the variables with mortality *via* the univariate and multivariate analyses is shown in **Table 3**. The univariate analysis identified the variables, including BISAP ≥ 3, SAP, MDR-GNB bacteraemia, and major complications, as predictors of mortality. MDR-GNB bacteraemia (OR = 8.976, 95% CI = 1.805 –44.620, *p* = 0.007) and SAP (OR = 9.414, 95% CI = 1.742 –50.873, *p* = 0.009) were both statistically significant in the multivariate logistic regression analysis.

**Table 3:** Univariate and multivariate analyses of predictors associated with mortality in 72 IPN patients.

Variable, n (%)	Survival (n = 53)	Mortality (n = 19)	Univariate analysis		Multivariate analysis	
			OR (95% CI)	*p*	OR (95% CI)	*p*
BISAP ≥ 3	15 (28.3)	14 (73.7)	7.093 (2.173–23.157)	0.001*	1.779 (0.337–9.389)	0.497
Male	43 (81.1)	13 (68.4)	0.504 (0.154–1.651)	0.504		
SAP	12 (22.6)	16 (84.2)	18.222 (4.535–73.220)	<0.001*	9.414 (1.742–50.873)	0.009*
MDR-GNB bacteraemia	9 (17.0)	15 (78.9)	18.333 (4.919–68.223)	<0.001*	8.976 (1.805–44.620)	0.007*
Major complications	18 (34.0)	13 (68.4)	4.213 (1.372–12.938)	0.012*	1.736 (0.360–8.375)	0.492
Concomitant infections	11 (20.8)	7 (36.8)	2.227 (0.709–6.995)	0.170		

Note. IPN, infected pancreatic necrosis; MDR-GNB, multiple drug-resistant Gram-negative bacterial; BISAP, bedside index of severity in acute pancreatitis; SAP, severe acute pancreatitis.

*P values are statistically significant.

### Distribution of multiple drug-resistant Gram-negative bacterial strains

**Figure 2:**
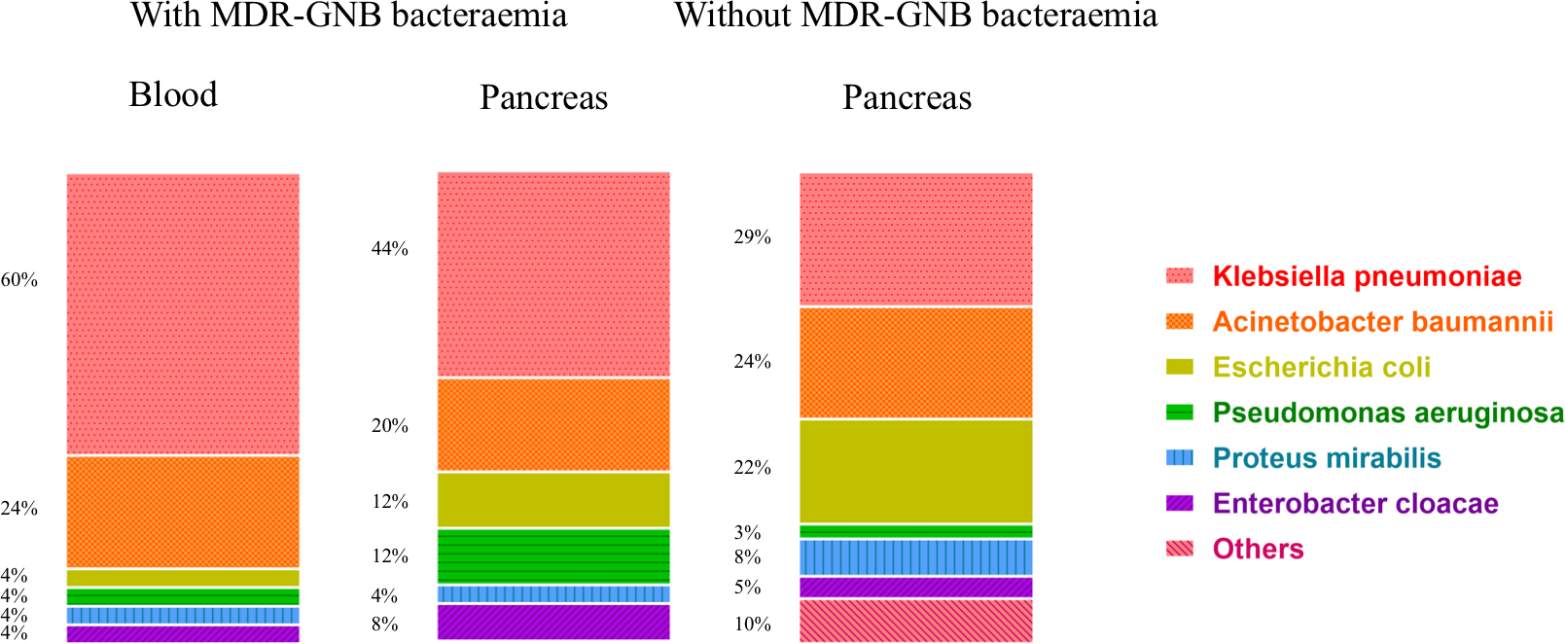
Distribution of 113 MDR-GNB strains among 72 IPN patients with and without bacteraemia. MDR-GNB, multiple drug-resistant Gram-negative bacterial; IPN, infected pancreatic necrosis.

In **Figure 2**, all 113 respective bacterial strains in 72 IPN patients were divided into with and without bacteraemia groups (a total of 50 and 63, respectively). In the pancreas, *Klebsiella pneumoniae* is the most common species in both with (11 of 25) and without MDR-GNB bacteraemia (18 of 63) followed by *Acinetobacter baumannii* (5 of 25 and 15 of 63, respectively) and *Escherichia coli* (3 of 25 and 14 of 63, respectively). In the blood, *K. pneumoniae* is also the primary bacterium (15 of 25).

## Discussion

The emergence of MDR-GNB is increasingly becoming a major and global public health issue with rising antibiotic resistance and increased mortality (Mills and Marchaim, 2021). The incidence of bacteraemia due to MDR-GNB is higher than that of other infections (Diekema et al., 2019). Uncontrollable bacteraemia, secondary to AP, may play a key role in this prognosis. This is the first case–control study that has focused on the impact of MDR-GNB bacteraemia on IPN patients, and the mortality of IPN in this study was up to 26.4% in accordance with previous studies, which highlighted that MDR-GNB has become a serious pathogen of infections among AP patients (Jain et al., 2018; Firsova et al., 2020).

We found that MDR-GNB bacteraemia resulted in higher mortality and longer ICU stays and was related to higher BISAP scores at admission, SAP, and biliary AP. In line with Li et al., hyperlipidaemia was the primary aetiology in the IPN patients, but it was interesting that the biliary aetiology was more frequent in patients within the MDR-GNB bacteraemia group than the contrast group, thus suggesting that MDR-GNB bacteraemia could be secondary to bacterial translocation through the gut (Flint and Windsor, 2003; Li et al., 2020; Yang and McNabb-Baltar, 2020). In addition, these invasive treatments may result in bacteraemia, especially MDR-GNB bacteraemia (Wu et al., 2021b). In our study, there were also 12 of 24 patients diagnosed with bacteraemia during the post-intervention (≤5 days), which may be possibly related to translocation secondary to intervention and needs to be validated in the larger sample size study. According to Hagjer et al., a high BISAP score, similar to SAP, indicated more severe OF, which needed invasive treatments, such as ventilators, central venous catheter support, and hemodialysis (Hagjer and Kumar, 2018). However, bacteraemia, especially sepsis shock, could worsen OF and mimic SAP. In the subgroup comparison (moderately SAP vs. SAP) in the MDR-GNB bacteraemia, the mortality was significantly increased by the severity of AP, which was also accompanied by high procalcitonin in accordance with previous studies investigating markers of severe infections (Lin et al., 2017; Hong et al., 2019; Wu et al., 2021c; Wu et al., 2022b). It is worth noting that MDR-GNB bacteraemia had a mortality rate of 62.5% resultant from untimely detection and inappropriate antibiotic treatment, which has also been revealed to be associated with a greater risk of hospital mortality among critically ill patients with bacteraemia (Diekema et al., 2019). We revealed that both MDR-GNB bacteraemia and SAP were independent predictors of overall mortality in multivariate analysis, which suggested that bacteraemia plays a key role in the severity of AP.

For the distribution of pathogens, in accordance with previous studies, MDR-*K. pneumoniae* was revealed to be the most common pathogen of IPN, which enhances the theory of colonizing bacterial translocation (Tugal et al., 2015; Wu et al., 2021c). Interestingly, MDR-*K. pneumoniae* was also the primary species in the blood. However, this must be further validated in order to support the hypothesis that bacteria, released from invasive intervention and gut, may enter the bloodstream. The timing of MDR-GNB bacteraemia was not as in other studies, which suggests that extra-pancreatic infections develop before IPN (Lu et al., 2019; Garret et al., 2020). This may possibly be due to the delayed intervention in addition to a step-up strategy for IPN and untimely bacteraemia diagnosis (Boxhoorn et al., 2021). Routine bacterial culture, which relies on the number of bacteria in the specimen, was not sensitive enough in establishing the microbiological bacteraemia diagnosis without a specific clinical presentation different to the severity of AP itself (Carey et al., 2011). Novel molecular and phenotypic rapid tests for identification and antimicrobial susceptibility testing are recommended as one of the best choices for MDR-GNB bacteraemia, which reduce both the time of treatment and the misuse of antibiotics (Giacobbe et al., 2020). The problem of overuse of broad-spectrum antibiotics, especially carbapenem or third- generation cephalosporin β-lactams, has not been solved in line with the latest guideline (Baron et al., 2020). MDR-GNB screening, especially MDR or carbapenem-resistant *K. pneumoniae*, at ICU admission is considered an effective approach for the surveillance of MDR-GNB colonization in the high-incidence area, guiding clinicians to prescribe appropriate antibiotics in AP patients complicated with “suspected” bacteraemia when blood culture results are pending. In this era with the MDR bacteria, we must pay attention to protecting organ function and reducing invasive treatment or ICU stays instead of creating or overusing “new” antibiotics. Prevention is more important than treatments for MDR-GNB bacteraemia among AP patients.

Although many advances in understanding the role of MDR-GNB bacteraemia in AP patients have taken place in this study, there are also some limitations. Firstly, our study is limited to deficient variables and potential selection biases by its retrospective case–control nature. We performed the latest precise definition and criteria to decrease the suboptimal control selection biases, which may result in only a very small reduction in power. Secondly, the limited sample size of the case group reduced the power of risk analyses, as indicated by the wide range of confidence intervals, which is a common problem in studies identifying risk factors for mortality due to MDR organisms. Thirdly, the surgical aspects of IPN, including locations and subsequent choices of interventions, were not discussed due to the specific focus on the impact of bacteraemia. Fourthly, the percentage of SAP was significantly higher in the MDR-GNB bacteraemia group. The OF caused by both bacteraemia and the severity of AP could not be ignored, and SAP patients could suffer from more exposure to antibiotics and ICU stays, which would possibly result in MDR-GNB bacteraemia. There may be a mutual cause-and- effect relationship between the severity of AP and MDR-GNB bacteraemia, which may be hard to correct in multivariable analysis. Thus, we could only analyze the overall mortality, instead of infection- related mortality, which needs to be further investigated in the future. Finally, we chose the IPN patients without MDR-GNB bacteraemia as the control group and used concomitant infections as a variable, so our study could only assess the hazard of MDR-GNB bacteraemia on the IPN patients instead of patients who suffered from other infections. This effect needs to be further investigated.

## Conclusion

Higher mortality and the possibility of SAP were observed in patients with MDR-GNB bacteraemia, and further efforts to prevent MDR-GNB bacteraemia are urgently needed. SAP combined with MDR-GNB bacteraemia could significantly increase mortality. SAP and MDR-GNB bacteraemia were two independent predictors of mortality in IPN patients. MDR- *K. pneumoniae* was the most frequent pathogen in both blood and pancreas, and this merits further attention in future studies.

## Data availability statement

The raw data supporting the conclusions of this article will be made available by the authors, without undue reservation.

## Ethics statement

The studies involving human participants were reviewed and approved by Institutional Review Board of Xiangya Hospital. Written informed consent for participation was not required for this study in accordance with the national legislation and the institutional requirements. Written informed consent was not obtained from the individual(s) for the publication of any potentially identifiable images or data included in this article.

## Author contributions

DW and YJ contributed equally to this article. DW and YJ participated in the design of the study and drafted the manuscript. WC, YH, AK, DL, and RS participated in data collection and analysis. JP conceived of the study, and participated in design and coordination and helped to draft the manuscript. All authors contributed to the article and approved the submitted version.

## Funding

This study was supported by Fundamental Research Funds for the Central Universities of Central South University (Grant Nos. 2021zzts0347 and 2021zzts0352), Hunan Provincial Innovation Foundation for Postgraduate (Grant No. CX20210357), China Scholarship Council (Nos. 202106370184 and 201806240274), and National Natural Science Foundation of China (Grant Nos. 81670589 and 82170661).

## Acknowledgments

We thank Dr. Xiexiong Zhao for his work on language editing and Prof. Junxia Yan for her work in the verification of statistics.

## Conflict of interest

The authors declare that the research was conducted in the absence of any commercial or financial relationships that could be construed as a potential conflict of interest.

## Publisher’s note

All claims expressed in this article are solely those of the authors and do not necessarily represent those of their affiliated organizations, or those of the publisher, the editors and the reviewers. Any product that may be evaluated in this article, or claim that may be made by its manufacturer, is not guaranteed or endorsed by the publisher.
